# The Curse of the Chimney

**Published:** 1909-03-20

**Authors:** 


### THE CURSE OF THE CHIMNEY.

Every winter, with its inevitable fogs and phalanx of catarrhal complaints, draws strong attention to the necessity that exists in English cities for enforcement of the laws which have been enacted to stem, though in a trivial and small degree merely, the evil of our gigantic smoke nuisance. On more than one occasion we have commented on the laxity with which these laws and regulations—admittedly excellent if carried out in the strict letter as well as spirit—are enforced. Every winter compels us to return to the subject, and to support, as much as it lies in the power of a professional paper, the efforts of the Anti-Smoke Society to mitigate the evil.

The medical profession, as a collective body, has been singularly apathetic in moulding public opinion on this subject of city smoke and its bad consequences. Yet we, as medical men, are thoroughly well aware of the danger to health that exists in the continuance of fogs and a soot and smoke impregnated atmosphere. There are few of us who are conservative enough or quasi-scientific enough to believe that the continual inspiration of much diluted sulphuric acid and a cloud of carbon particles can strengthen anybody's constitution or act otherwise than detrimentally on the economy of anybody's lungs. There is still, we believe, an absurd impression lingering in the minds of a small section of the community that constant exposure to fogs, especially to those of the London pea-soup order, has a tendency to "invigorate" the organism. We have even heard this theory enunciated by those who claim to find it supported in the returns of the Registrar-General, and point to the comparatively low mortality which smoke-ridden cities are alleged to enjoy. Such considerations, however, cannot weigh with anyone, professional or layman, who is aware of the evil effects of smoke and soot upon the vegetable and animal life of large cities. There is no need to enumerate these results here. It is only worth while to point out that fogs are not entirely negligible factors in the causation of many varieties of pulmonary, nasal, throat, and aural lesions, and to lay stress on the fact that the evil that they do is as extensive (and as little to be put in the form of bald statistics) from a hygienic point of view as from the commercial.

That fogs are, if not wholly preventable, then at least to a large extent to be decreased in severity by proper prophylactic measures, is an axiom that may be laid down without fear of much contradiction. There are admirable examples of what due enforcement of anti-smoke laws and adequate combustion methods can do. The city of New York, which is singularly free from fog of the London variety, is a proof of this fact. There the use of smoky coal is prohibited by law: hard coal is generally used, and effective combustion apparatus is fixed to each furnace and chimney flue. The smoke that issues from the stacks and curls over the sky-scrapers is white, like cirrus cloud, and with the exception of small whorls of grey-black, emanating from engine funnel or house chimney, it is rare to find a column of smoke of the deadly carbonaceous hue that is so familiar in London, Manchester, Liverpool, and, in fact, in all our large cities. What is being done in America can as easily be achieved here. That it will be achieved in time there is no doubt whatever in our minds.

It is unbelievable that the public will go on, year after year, tolerating a state of things which it is good economy to prevent, and bad policy, from a hygienic point of view, to permit. Meanwhile it rests as seriously with the medical profession as with members of especialised societies to impress upon the community the desirability of exerting themselves in this matter as soon as possible. The case against the fog is a strong one; the case against the smoking chimney as one of the factors in the causation of fogs is yet stronger. So long as the laws against the smoke nuisance are allowed to remain dead letters it is useless to debate the feasibility of expensive and problematical measures such as the electrical dissipation of fog, or the yet more problematical remedy of fog dispersal by means of artificially created air currents. The remedy lies at hand in the due enforcement of existing regulations. When once these are thoroughly carried out the annual toll that our cities pay to the fog fiend—in lives, in money, in comfort, and in health, will inevitably diminish to a material extent.

